# Methodology for Definition of Yellow Fever Priority Areas, Based on Environmental Variables and Multiple Correspondence Analyses

**DOI:** 10.1371/journal.pntd.0001658

**Published:** 2012-07-03

**Authors:** Eduardo Stramandinoli Moreno, Rita de Cássia Barradas Barata

**Affiliations:** Santa Casa of São Paulo School of Medical Sciences, São Paulo, Brazil; Centers for Disease Control and Prevention, United States of America

## Abstract

Yellow fever (YF) is endemic in much of Brazil, where cases of the disease are reported every year. Since 2008, outbreaks of the disease have occurred in regions of the country where no reports had been registered for decades, which has obligated public health authorities to redefine risk areas for the disease. The aim of the present study was to propose a methodology of environmental risk analysis for defining priority municipalities for YF vaccination, using as example, the State of São Paulo, Brazil. The municipalities were divided into two groups (affected and unaffected by YF) and compared based on environmental parameters related to the disease's eco-epidemiology. Bivariate analysis was used to identify statistically significant associations between the variables and virus circulation. Multiple correspondence analysis (MCA) was used to evaluate the relationship among the variables and their contribution to the dynamics of YF in Sao Paulo. The MCA generated a factor that was able to differentiate between affected and unaffected municipalities and was used to determine risk levels. This methodology can be replicated in other regions, standardized, and adapted to each context.

## Author Summary

Yellow fever (YF) is an infectious disease, transmitted by mosquitoes, and very common in North and Middle East region of Brazil, where cases of the disease are reported every year. Since 2008, outbreaks of the disease have occurred in regions of the country where no reports had been registered for decades, which has obligated public health authorities to redefine risk areas for the disease. The aim of the present study was to propose a methodology of environmental risk analysis for defining priority municipalities for YF vaccination. The municipalities were divided into two groups (affected and unaffected by YF) and compared based on environmental parameters related to the disease's epidemiology. Statistical analysis was used to identify associations between the variables and virus circulation, as well as, to evaluate the relationship among the variables and their contribution to the dynamics of YF. The MCA generated a factor that was able to differentiate between affected and unaffected municipalities and was used to determine risk levels. This methodology can be replicated in other regions, standardized, and adapted to each context.

## Introduction

Brazil has an extended enzootic or endemic area for sylvatic yellow fever (YF), where cases of the disease are annually reported. The highest frequency of the disease occurs between January and April, when high levels of rainfall and an increase in the vector population coincide with greater agricultural activity [1]–[5].

In Brazil, endemic cases of the disease were limited to the northern, middle, western, and pre-Amazon regions until 1999 [4], [6]. Since then, YF has progressively expanded its territory, and a gradual increase of reported cases has been observed near the traditional boundaries of endemic zones. This expansion highlights the need to redefine the areas of risk [6]–[9].

Until 2008, four distinct epidemiologic area types for YF were acknowledged in Brazil: endemic areas (where vaccination against YF was recommended), transition areas (also known as epizootic or emergence areas), potential risk areas (where vaccination against YF was not recommended) and disease-free areas (where YF did not occur and vaccination against YF was not recommended) [4], [6]. Zones classified as —transition, and —potential risk, have no records of virus circulation and no indication for YF vaccination. However, these areas possess some environmental parameters that are compatible with the establishment and maintenance of the disease; thus, there was a need for increased YF surveillance activities in those regions. Nevertheless, these parameters were subjectively defined and the non-vaccination of supposedly at-risk people generated ethical problems for Brazilian health authorities.

The transition and potential risk zones were eliminated in 2008. Therefore, only two area types are currently acknowledged: endemic (where vaccination against YF is recommended) and disease-free (where vaccination against YF is not recommended).

In public health emergency situations, the municipalities where vaccination should be recommended are defined by classification methods based on affected or expanded areas. Thus, municipalities are considered to be affected when the virus circulation can be detected, which occurs when YF epizooties are confirmed in nonhuman primates, when there are confirmed human cases, or when the virus is isolated in mosquitoes [6], [7]. Municipalities within 30 km of a municipality where virus circulation has been detected are also considered to be affected areas [6].

The YF vaccine was considered completely safe until 2001, as there had been no reports of serious adverse reactions associated with its administration. However, 12 serious cases were reported in 2001 [10]–[12], and 39 additional cases were identified worldwide through May of 2009 [13]; to date, over 50 cases have been reported [13]–[15]. Two types of serious adverse reactions are commonly reported: neurotropic disease, which is caused by the invasion of the nervous system by the vaccine virus, and viscerotropic disease, which is a pan-systemic infection that is similar to the infection caused by the wild-type virus [13].

A dilemma is thus created for the public health authorities: what proportion of the at-risk population should be vaccinated to minimize the total number of fatal cases from the natural infection of the yellow fever virus (YFV) or the vaccine virus?

This problem applies to the State of Sao Paulo and to other states located in the southern and southeastern regions of Brazil. Briand *et* al. (2009) [16] developed a methodology for prioritization of areas for vaccination against YF for countries in Africa, using Multiple Correspondence Analysis. Although, in this study the authors had as limitation: the lack of information available, working with a small number of variables.

Using the current situation of the State of Sao Paulo, Brazil, as an example for definition of priority areas for vaccination against YF, this paper aims to adapt the methodology of risk analysis proposed by Briand *et* al, (2009) [16] in a context with more availability of information, allowing the use of environmental variables potentially related with the eco-epidemiology of YF.

## Methods

The study was conducted in the State of Sao Paulo, Brazil. Sao Paulo is composed of 645 municipalities, has an area of approximately 250,000 km^2^, and has an estimated population of 40 million people. There are currently 429 municipalities in the YF-endemic zone and 216 in the disease-free zone.

Aiming to select variables with the most relevance for the eco-epidemiology of YF, two groups were defined for comparison: municipalities that were affected and municipalities that were unaffected by the disease. The study used a case-control model with an ecological approach.

The Text S1 shows the resume of the steps used in this study. Municipalities with confirmed YFV circulation in their territory and the adjacent municipalities were considered to be affected [6]. There were a total of 12 municipalities with confirmed YFV circulation and 57 adjacent municipalities. The 12 confirmed municipalities and 18 randomly selected adjacent municipalities were included in this study and constituted a sample of 30 cases, which is the minimum necessary for the use of the desired statistical analysis.

**Figure 1:**
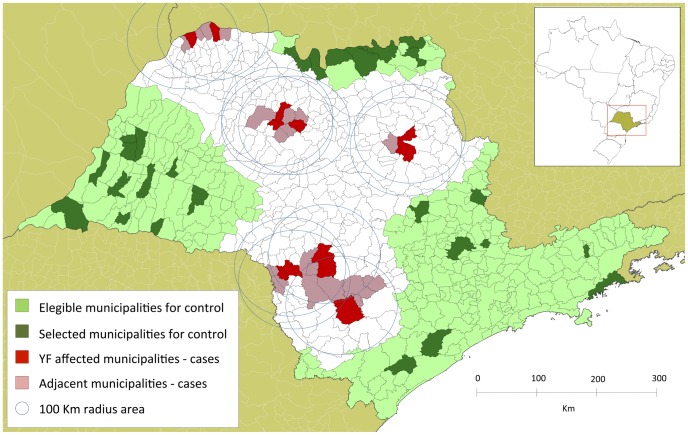
YF-affected and unaffected municipalities selected for the present study, São Paulo, Brazil.

The unaffected or eligible control municipalities consisted of all of the municipalities that had no reported cases of YF and that were at least 100 km away from any affected municipality. Figure 1 illustrates the methods used to outline these areas and the municipalities selected for this study.

Each municipality was analyzed relative to the moment before the occurrence of YF in its region or prior to its inclusion as an area of recommended vaccination. Following Briand *et* al. (2009) [16], the variables were selected to relate to risk allocation based on vulnerability according to three main axes: exposition, susceptibility, and resilience. The authors considered exposition as the capacity for YFV to circulate in a municipality. Thus, data included were related to the environment (land occupation, forest fragmentation, wind direction influences, distance for biodiversity conservation unities, distance for municipalities with YFV circulation and proportion of riparian forest), the vectors (temperature, humidity and pluviosity), and the hosts (human displacements and illegal animal trafficking).

Susceptibility was considered as the number of hosts without immunity for YFV that lived in each municipality. The immune human population was calculated as the proportion of immunized people divided by total population of the municipality. Non-human primates comprised the registered species occurrence in each municipality classified by a score according with the importance of each species as YFV amplifier [17]. Susceptibility also included the risk of urbanization of the disease, based on levels of infestation of *Aedes aegypti* in the municipality, using the Breateau Index [18].

Resilience was defined as the capacity of each municipality to detect the YFV circulation in its territory (Surveillance for Febrile Ictero-hemorrhagic Syndrome), as well as, its capacity of confrontation an outbreak of YF (Medical care capacity).

The Text S2 shows the variables analyzed in the study. The secondary data were primarily obtained from the Internet. The free software *Terraview* 3.3.1 was used for distance measurements. Historical series were created for the variables temperature, pluviosity, and humidity using the monthly averages from November to May (months with a greater occurrence of YF). The mean pluviosity divided by the mean real evapotranspiration (RET) in the same period was used as a humidity indicator [19].

Variables that showed statistically significant associations (chi-squared test, p<0.05) were selected for the multiple correspondence analysis (MCA). For the application of MCA, all the variables were categorized and treated like qualitative variables. The MCA is an exploratory and descriptive multivariate statistical technique for categorical data analysis. The technique is appropriate for the analysis of contingency tables with a large number of variables. The method analyses the mass distribution, by the pattern of the frequency for the considered categories, aiming to identify the uniformity of the distribution. This analysis was performed to evaluate the relationships among the selected variables and to obtain factors that best represent all variables, considering the level of significance (weight) of each to explain total sample variability (inertia). Thus, the graphic obtained can be studied like a geographic map, analyzing the relationship of proximity by projections of the factors, in way that each point represents each variable. *STATISTICA 7* software was used to perform the MCA.

## Results

The bivariate analysis identified seven variables associated with YFV circulation (Table 1). The MCA generated 12 factors to explain the total sample variability (inertia). One of the factors could independently explain 28.1% of the total sample variability. None of the other 11 factors were able to independently explain more than 10% of the sample variability.

**Table 1:** Statistically significant variables related to YFV circulation when comparing the two groups.

Variable	*p-value*
Distance to area with recommended vaccination against YF	0.007
Distance to a biodiversity conservation unit	0.01
Influence of the direction of dominant wind routes	0.0007
Proportion of riparian forest	0.0008
Number of main routes of illegal wildlife traffic up to 100 km away	<0.0001
Humidity (Pluviosity/RET)	<0.0001
Surveillance for Febrile Ictero-hemorrhagic Syndrome (SFIHS)	<0.0001

Variables associated with YFV circulation in the State of Sao Paulo.

**Figure 2:**
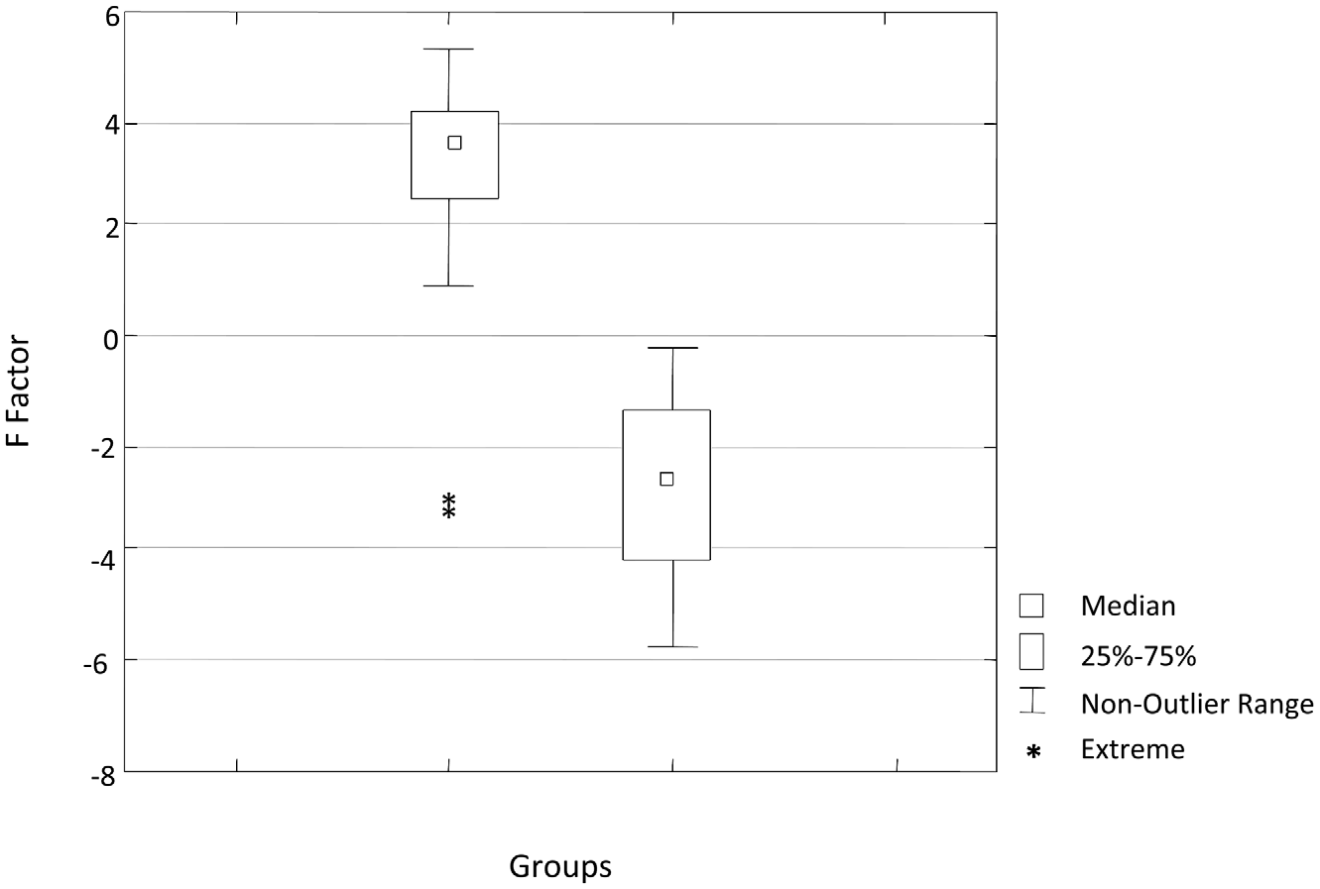
Distribution of variables used in the MCA according to their contribution to the F factor. (DIST_VAC = Distance to areas with recommended YF vaccination; DIST_UC = Distance to a biodiversity conservation unit; MATA = proportion of riparian forest; TRAF = Number of main routes of illegal wildlife traffic up to 100 km away; HUMID = Humidity (Pluviosity/RET); VENT = Influence of the direction of dominant wind routes; SFIHA = Surveillance for Febrile Ictero-hemorrhagic Syndrome (SFIHS).

The analysis of the graphic (Figure 2) allows visualization of the relationship between the variables used for the construction of F factor. The graph contains only one dimension, and each point's disposition represents the position of each variable.

Three main clusters of variables could be noted. The first cluster shows a collection of variables that represent, in theory, lower risk for the occurrence of YF. These variables are represented by extreme values: greater distances to areas with recommended vaccination against YF (DIST_VAC:1) and biodiversity conservation units (DIST_BCU:1), smaller proportions of riparian forest (RIPA:3), fewer routes of illegal wildlife traffic (TRAF:3), less humidity (HUMI:1), less influence of the direction of dominant wind routes (WIND:3), and no surveillance for SFIHS (SFIHS:2).

The second cluster shows variables of intermediate values and the third shows opposite values of those observed in the first cluster. The variable distance to area with recommended vaccination against YF was the only exception observed, where values for —adjacent or up to 30 km (DIST_VAC:1) and —31 to 100 Km (DIST_VAC:2) were clustered between the first and second clusters.

The weights of each variable (Table 2) were identified by MCA, based on geometric distance between than in the graphic. Thus, these values were used on the equation, and the F factor was calculated for each municipality.

**Table 2:** Weight of each variable for F factor calculation.

Variables	Weight
Distance to area with recommended vaccination against YF – up to 30 km	0.306264
Distance to area with recommended vaccination against YF – 31 to 100 km	0.244947
Distance to area with recommended vaccination against YF – over 100 km	−0.78091
Distance to biodiversity conservation unit – up to 30 km	0.648733
Distance to biodiversity conservation unit - 31 to 100 km	0.013377
Distance to biodiversity conservation unit – over 100 km	−1.06924
Proportion of riparian forest – up to 30%	−0.71405
Proportion of riparian forest – 31 to 60%	−0.24182
Proportion of riparian forest – 61 to 100%	0.932843
Number of main routes of illegal wildlife traffic – Low	−0.85655
Number of main routes of illegal wildlife traffic – Medium	−0.65112
Number of main routes of illegal wildlife traffic – High	1.087954
Influence of the direction of dominant wind routes – Low	−0.63524
Influence of the direction of dominant wind routes – Medium	0.073681
Influence of the direction of dominant wind routes – High	1.123113
Humidity – (Pluviosity/RET) – smaller than 1.5	−0.93357
Humidity (Pluviosity/RET) – greater than 1.5	0.400103
Surveillance for SFIHS – Yes	−0.64806
Surveillance for SFIHS – No	0.847463

It's known that municipalities without the SFIHS are less resilient. So, the association of this variable with the YFV circulation was considered as protection factor. Thus, the positive sign of the variable was inverted for the calculation of the F factor, in way that, municipalities without SFIHS had its F factor increased, and so, considered more vulnerable.

**Figure 3:**
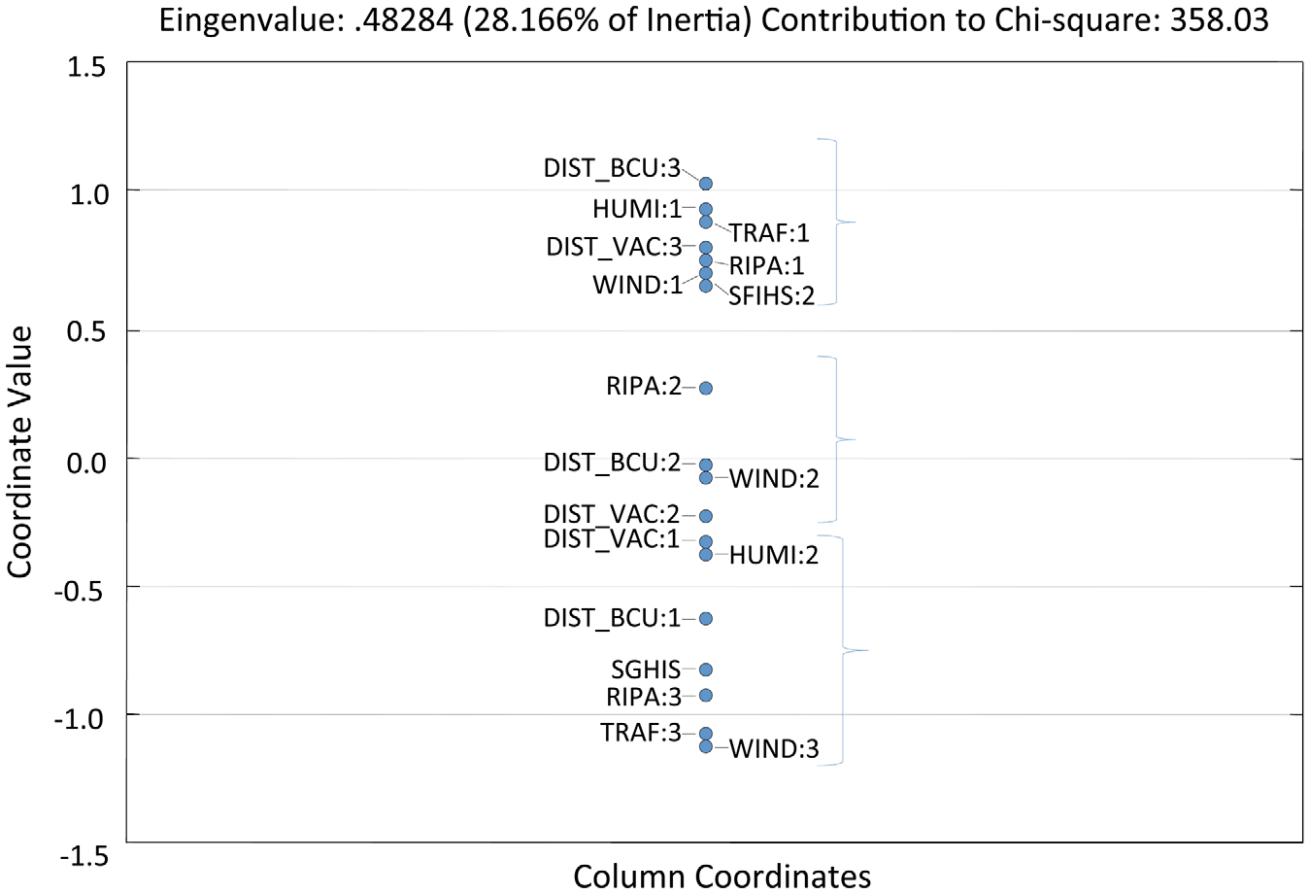
Distribution of the evaluated groups of municipalities according to the F factor, State of São Paulo, Brazil. (Group 1 – YF-affected municipalities; Group 2 – unaffected municipalities).

Analyzing the graph (Figure 3) allows us to observe the difference between cases group 1) and controls (group 2) according to the F factor. All municipalities from control group (non-affected) showed values under zero. So, this was defined as the cut point to differentiate risk and no risk. The scale of risk was divided in two to turn the model able to give priority for municipalities with higher F factor values.

Thus, the priority levels for vaccination against YF in municipalities of the State of Sao Paulo were: F factor<0.0 = low risk; 0.0<F factor<2.0 = some risk; F factor>2.0 = high risk.

## Discussion

The study used a large number of variables. Much of the work focused on the collection and standardization of the information from secondary sources (i.e. the internet). The 60 municipalities evaluated in the case-control step was the minimum necessary to support the statistical analysis. The State is composed of 645 municipalities, thus, the collection of all information for each municipality would be unfeasible. Therefore, effort was made to identify the most important variables. This approach, using a subset of municipalities, simplified and optimized the method for broader application to target municipalities with not currently indication for YF vaccination.

The use of secondary data that are available on the Internet is one limitation of this methodology, especially given that the data were not collected for this purpose. However, the authors sought to incorporate official published data on each subject. Therefore, the limitation is admitted for a better replicability of the method.

The study showed the importance of a large number of variables for the ecoepidemiology of YF.

The distance between the municipality and areas with recommended vaccination against YF can be considered an important criterion for the prioritization of a municipality for YF vaccination. Municipalities from affected regions were, for the most part, close to or even inside of areas with recommended vaccination against YF at the moment of the case occurrence. The occurrence of YF in municipalities with a small proportion of susceptible individuals indicates the importance of vaccination coverage of close to 100% for populations living in areas of risk, as is recommended by Brazil's Ministry of Health [6].

Municipalities located in affected regions were closer to biodiversity conservation units (BCU). Mosquito species that serve as vectors for YF are mainly found in well-conserved forest patches [20]. It is possible that BCUs favor the proliferation of these species and increase the chances of disease maintenance by serving as stepping stones for the geographic expansion of the disease.

The Brazilian Forest Code includes the riparian forests in the category of permanently protected areas. Thus, forest patches are more frequently maintained in these environments and generate more stable ecological corridors. These forests represent one of the few environments that allow the displacement of non-human primate populations.

Affected municipalities were closer to main illegal wildlife traffic routes. Trafficking of illegal wildlife can be an important source for the dissemination of viremic non-human primates from areas of virus circulation. Every year, large numbers of non-human primates that originate from YF-endemic regions, such as the Amazon, are apprehended from illegal trafficking [21]. These animals are often returned to forest environments without adequate ecological and sanitary evaluations, which allows for contact between these viremic hosts and vectors of the disease [22].

Climatic factors, such as humidity and temperature, have a direct influence over the abundance of YF mosquito vector species, as well as virus multiplication in its arthropod reservoirs [23]–[26]. Unlike the humidity calculated as a percentage relative to the availability of water vapor in the air, the RET is calculated in mm^3^, which allows for the evaluation of its relationship with the pluviosity and hydrologic balance of the region. Given that the RET takes into consideration several factors, it is a more complete indicator of climatic conditions than the isolated values of pluviosity and temperature; therefore, it better represents the context of topography and land occupation in the municipalities [19], [27].

Another climatic factor that presented a statistically significant association between the groups was the influence of dominant wind routes that arrive at each municipality. The biological plausibility of this hypothesis is related to the possibility of dispersion of mosquito vectors by dominant winds [28]–[31]. Causey *et* al. (1950) [29] evaluated the dispersion patterns of mosquitoes of the genus *Haemagogus spp.* And *Sabethes spp.* in the State of Minas Gerais, Brazil. Dispersion capabilities of up to 11 km were observed. The authors concluded that environments composed of forest patches, agriculture, and pasture favor the expansion of YF by increasing the wind dispersion of mosquitoes.

The importance of active Surveillance for Febrile Ictero-hemorrhagic Syndrome (SFIHS) was also demonstrated by the present study. The affected municipalities mostly coincided with regions of the state where this surveillance system had been implemented. Therefore, municipalities without SFIHS had less resilience, meaning a lower capacity for disease detection to address a possible virus circulation in its territory.

Due to the large number of important variables for YF eco-epidemiology in this study, it is possible to visualize the complexity of the disease. Several factors probably act simultaneously and in different combinations to determine virus establishment and maintenance in a region. Therefore, multivariate analysis techniques are important for the evaluation of the influence of each variable on the disease's eco-epidemiology.

Variables that showed greater contributions to the variability of the municipalities in relation to the F factor observed in this study were influence of the direction of dominant wind routes, number of illegal wildlife traffic routes, proportion of riparian forest, and the implantation of surveillance of FIHS.

The grouping of the variable distance to area with recommended YF vaccination into groups of —up to 30 km, and —31 to 100 km, suggests a possible need to increase the current 30-km radius for the areas considered to be at risk (expanded areas) during outbreaks of YF.

The legislation that establishes the YF surveillance system in Brazil [6] defines that this System must be based on confirmed cases rather than predictions about the occurrence of the disease in areas of potential risk. The main purpose of the system is focused on the rapid detection of suspect cases and the adoption of emergency measures that will prevent an epidemic outbreak. The previous approach for risk classification of YF in Brazil, using —transition, and —potential risk, areas for guiding control measures, allowed for the intensification of surveillance in areas of known environmental potential for disease establishment. However, this approach was highly subjective because the criterions for defining areas were not described in a systematic way. The difficulty in replicating courses of action led to the simplification that is the current method [32].

However, it is extremely important that a surveillance system, such as that for YF—a fatal disease with great potential for outbreaks—works with models for supporting an evidence-based public-health decision-making process to guide actions in outbreak emergency situations. In the long run, the goal is to interrupt the expansion of the disease to large populated areas or known vulnerable populations. The present study has proposed a methodology for the definition of vulnerable regions for YF using environmental variables and a systematic design that focuses on a regional scale.

The difference between the current method and the method proposed can be noted by the fact that all control municipalities, which were located in area with YF vaccination indicated, but without registration of YFV circulation, were classified as without risk in this study [6], [32].

In this sense, it is recommended that, within the vaccination, municipalities classified as in —risk, pass through an analysis of its structural capacity for confrontation of a YF outbreak, considering: the number of technicians trained and sensitized for: detection of YF suspected cases, treatment and laboratorial diagnosis resources, viability for detection of epizootic events in non-human primates, capacity for conduction of entomological studies, and viability for timely conduction of campaigns of vaccination for target populations when required.

In the case of municipalities classified as “high risk”, it is recommended that, in addition to the measures cited above, the organization of surveillance system for SIHFS be conducted, once this system increases the sensitivity of the Yellow Fever Surveillance System in other regions of State affected by the disease [32].

It is also recommended that professionals using this methodology visualize the geographic distribution of municipalities according with the risk classification. This type of approach can be useful for organization of the action measures for disease control. Moreno & Barata (2011) [32] showed that, in Sao Paulo State, the municipalities with higher risk are the most populated. In cases like these, the increase of surveillance measures can be an option more feasible both financially as well as operationally.

The increased geographic expansion of emergent diseases, such as YF, exposes the health surveillance systems to the need to seek methodologies with multidisciplinary approaches that are able to adapt to different regional realities. Using locally relevant environmental variables and a systematic design, the methodology proposed in this study was able to differentiate municipalities according to their vulnerability for the occurrence of YF. This methodology can be replicated in other regions, standardized, and adapted to each context.

## Supporting Information

Text S1 **Resume of steps for development of the methodology.**
(DOC)

Text S2 **Variables and their respective data sources.**
(DOC)
