# Supplementary figures and images for: Methodology for Definition of Yellow Fever Priority Areas, Based on Environmental Variables and Multiple Correspondence Analyses
Source: PLoS Negl Trop Dis. 2012 Jul 3;6(7):e1658. doi: 10.1371/journal.pntd.0001658 (PMC3389021; doi:10.1371/journal.pntd.0001658)

**Supporting Text File 1 - Resume of steps for development of the methodology**


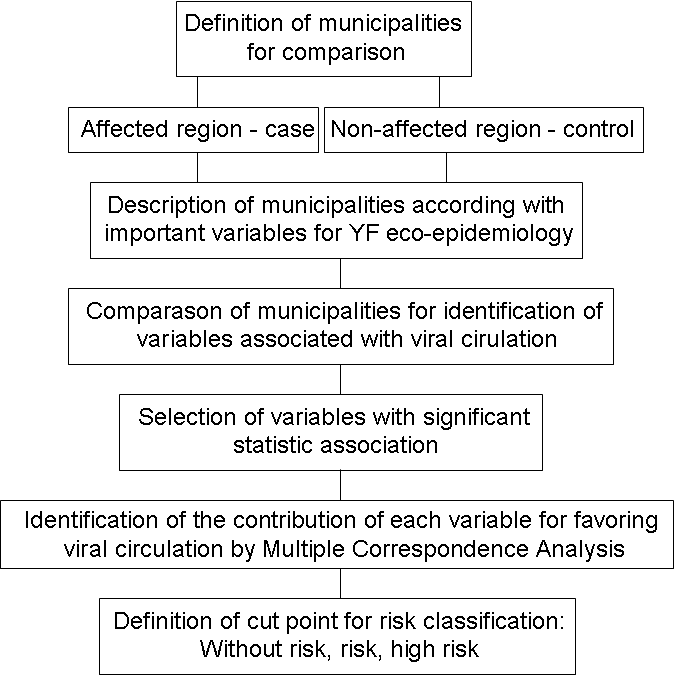

Supplement: Text S1 — Resume of steps for development of the methodology. (DOC) [file pntd.0001658.s001.doc]
