# Supplementary material for: Methodology for Definition of Yellow Fever Priority Areas, Based on Environmental Variables and Multiple Correspondence Analyses
Source: PLoS Negl Trop Dis. 2012 Jul 3;6(7):e1658. doi: 10.1371/journal.pntd.0001658 (PMC3389021; doi:10.1371/journal.pntd.0001658)
Supplement: Text S2 — Variables and their respective data sources. (DOC) [file pntd.0001658.s002.doc]

**Supporting Text File 2 - Variables and their respective data sources**

| **Variable** | **Data source** |
| --- | --- |
| **Land occupation** |  |
| - Proportion of natural vegetation and cultivated areas | Secretary of agriculture and livestock of the State of Sao Paulo, rural census of 2008. [www.cati.sp.gov.br/projetolupa/dadosmunicipais.php](http://www.cati.sp.gov.br/projetolupa/dadosmunicipais.php) |
| - Proportion of cultivated areas with a high demand for human resources |
| **Forest fragmentation** |  |
| - Main patch, patch density and mean patch size indexes | Forest Institute of São Paulo. [www.iflorestal.sp.gov.br/](http://www.iflorestal.sp.gov.br/) |
| **Proportion of riparian forest** | Forest Institute of São Paulo. [www.iflorestal.sp.gov.br/](http://www.iflorestal.sp.gov.br/) |
| **Influence of wind direction** | *Sérgio de Salvo Brito -* Reference center for Eolic and Solar Energy. [www.cresesb.cepel.br/index.php?link=/atlas_eolico_brasil/atlas.htm](http://www.cresesb.cepel.br/index.php?link=/atlas_eolico_brasil/atlas.htm) |
| **Distance to Biodiversity Conservation Unit** | Forest Institute of São Paulo. [www.iflorestal.sp.gov.br/](http://www.iflorestal.sp.gov.br/) |
| **Distance to area with recommended YF vaccination** | Technical reports (6-9) |
| **Temperature, Pluviosity and Humidity** | Integrated Center for meteorological information of the State of São Paulo. [www.ciiagro.sp.gov.br/sr_agromet.html](http://www.ciiagro.sp.gov.br/sr_agromet.html) |
| **Population displacement** | Road Department of the State of São Paulo [www.der.sp.gov.br/malha/estatisticas_trafego/estatisticas_trafego.aspx](http://www.der.sp.gov.br/malha/estatisticas_trafego/estatisticas_trafego.aspx) |
| - Mean number of cars that transit daily on the state’s main roads |
| - Distance to federal highway |
| **Distance to main routes of illegal traffic of wild animals** | Technical Report (20) |
| **Susceptibility of the human population** | National Immunization Program. <http://pni.datasus.gov.br/> |
| **Occurrence of non-human primate species** | Non-Human Primate Epizooties Surveillance  System – São Paulo State Health Department (17) |
| **YF Urbanization Risk – *Breteau* index** | Endemic Diseases Control Center. [www.sucen.sp.gov.br/gestor/baseda.html](http://www.sucen.sp.gov.br/gestor/baseda.html) |
| **Medical care capacity** | Health Department of the State of São Paulo. [www.saude.sp.gov.br/content/unidades_saude.mmp](http://www.saude.sp.gov.br/content/unidades_saude.mmp) |
| **Surveillance for SFIHS** | Technical Report (6) |
